# Flutamide Enhances Neuroprotective Effects of Testosterone during Experimental Cerebral Ischemia in Male Rats

**DOI:** 10.1155/2013/592398

**Published:** 2012-12-26

**Authors:** Hamed Fanaei, Hamid Reza Sadeghipour, Seyed Morteza Karimian, Gholamreza Hassanzade

**Affiliations:** ^1^Department of Physiology, Medical School, Tehran University of Medical Sciences, Enghelab Street, Tehran 1411413137, Iran; ^2^Department of Anatomy, Medical School, Tehran University of Medical Sciences, Enghelab Street, Tehran 1411413137, Iran

## Abstract

Testosterone has been shown to worsen histological and neurological impairment during cerebral ischemia in animal models. Cell culture studies revealed that testosterone is implicated in protecting neural and glial cells against insults, and they started to elucidate testosterone pathways that underlie these protective effects. These studies support the hypothesis that testosterone can be neuroprotective throughout an episode of cerebral ischemia. Therefore, we evaluated the mechanisms underlying the shift between testosterone protective and deleterious effects via block testosterone aromatization and androgen receptors in rats subjected to 60-minute middle cerebral artery occlusion. Fifty rats were divided into five equal groups: gonadally intact male; castrated male; intact male + flutamide; intact male + letrozole; intact male + combination flutamide and letrozole. Our results indicated that castration has the ability to reduce histological damage and to improve neurological score 24 hours after middle cerebral artery occlusion. Moreover, flutamide improved histologic and neurological impairment better than castration. Letrozole induced increases in striatal infarct volume and seizures in gonadally intact rats. Combination of flutamide and letrozole showed that letrozole can reverse beneficial effects of flutamide. In conclusion, it seems that the beneficial effects of flutamide are the prevention of the deleterious effects and enhancement of neuroprotective effects of testosterone during cerebral ischemia.

## 1. Introduction

Stroke is a great reason of disability and death throughout the world [1]. Nowadays treatments are not very effective to reduce brain ischemia, whereas size of the infarct area will affect on patient's chance of recovery from a stroke and it will keep growing if treatments are not appropriate [1–4]. So, for reducing damage we need to find more effective treatments. Among risk factors, sex has prominent role in stroke [5–8]. Epidemiological studies have shown that overall incidence of stroke is higher in men relative to age-matched women in most countries [9–12]. Present evidence suggests that mechanisms of cell death and neuroprotection are not similar and equal in males and females [9, 13, 14]. A large part of this difference between sexes is attributed to sex steroids [14–18]. Previous studies demonstrate that estrogen and progesterone give protection against cerebral ischemia by several mechanisms [19–23]. On the other hand, data about androgens are sparse and controversial [9, 18, 24, 25]. Human studies suggested male susceptibility to cerebral ischemia because male sex is a stroke risk factor in humans and low testosterone levels have been associated with risk for stroke and worse outcomes after stroke in men [9–12, 14, 17]. In animal studies, data are contradictory and show that androgens can protect or make worse cerebral injury [17, 26–29]. In rodents, testosterone replacement before cerebral ischemia in castrates increments histological injury [17, 26], whereas, stressors, such as halothane anesthesia, reduced brain injury, because when they applied before cerebral ischemia have potency to reduce plasma testosterone levels [30]. Moreover, testosterone replacement after an episode of cerebral ischemia accelerates histological and behavioral recovery in castrated rats [29]. These contradictory results may be reconciled by the hypothesis that testosterone has deleterious effects throughout an episode of cerebral ischemia but is beneficial in the recovery period. Differently in vitro studies showed both detrimental and beneficial effects of testosterone in neuronal cultures during exposure to different models of insult (e.g., oxidative stress, excitotoxicity, serum deprivation, and amyloid *β* toxicity) [26, 31–37]. One explanation for these inconsistent results of in vitro studies is that two potentially competing mechanisms exert beneficial and deleterious effects of testosterone during exposing to injury. These two possible mechanisms for testosterone can be androgen receptor (AR) dependent pathways or aromatization to estrogen and then activation of estrogen pathways [38, 39]. However, previous studies have not well determined effects of testosterone on cerebral cortex as well as striatum. None of these studies did whether the relation of testosterone and neurological deficit during the acute phase of cerebral ischemia has been mediated via AR or cerebral aromatase [9, 19, 38]. Accordingly, we aimed to investigate protective and/or deleterious effects of testosterone on cerebral ischemia of cortex and striatum as well as their contribution to neurological deficit in gonadally intact male rats subjected to transient middle cerebral artery occlusion (tMCAO). We used gonadally intact males experience tMCAO as a model of the effects of testosterone reduction during stroke. Moreover, flutamide (androgen receptor antagonist) and letrozole (aromatase inhibitor) were used to inhibit two potential mechanisms effects of testosterone.

## 2. Materials and Methods

All experiments were approved by Research Ethics Committees at Tehran University of Medical Sciences. All chemicals were obtained from Sigma-Aldrich (America), unless otherwise stated.

### 2.1. Animals and Treatments

Experiments were carried out on 50 male Wistar rats weighing 280–320 g. The rats were maintained under controlled conditions with temperature at 22–24°C, relative humidity of 50–60% and a 12-hour lighting cycle and permitted ad libitum access to water and standard lab chow. The experimental animals were divided into five groups (*n* = 10 per group) and each rat had 60 minutes tMCAO. Experimental groups were gonadally intact male; castrated male (castrations were operated 7 days before tMCAO); intact male + 10 mg/kg/sc flutamide; intact male + 1 mg/kg/i.p letrozole; intact male + 10 mg flutamide + 1 mg letrozole. Flutamide and letrozole were injected 2 hours before tMCAO. 

### 2.2. Determination of Serum Testosterone Concentration in Intact and Castrated Rats

Blood samples were taken from animals 48 h before and 24 h after tMCAO. Blood samples were centrifuged for serum separation and testosterone serum levels were determined by radioimmunoassay.

### 2.3. Transient Focal Cerebral Ischemia

tMCAO was induced by intraluminal filament method as previously described [40]. Briefly, animals were anesthetized with ketamine (100 mg/kg, i.p) and xylazine (10 mg/kg, i.p.) Right common carotid artery (CCA) and the external carotid artery (ECA) were exposed and carefully separated from the vagus nerve. tMCAO was produced by advancing a silicone-coated nylon monofilament through the ECA into the internal carotid artery (ICA) and then into the circle of Willis until a mild increase in resistance was felt. Such light resistance indicates that the tip of the nylon thread reached the origin of the MCA (18–20 mm from CCA bifurcation). Then, filament was fixed in place by fastening a silk suture around the ECA. Surgery was operated in 15 to 20 minutes. Sixty minutes later, the filament was withdrawn to allow brain tissue reperfusion. Rats were then allowed to recover and were observed according to the experimental protocol.

### 2.4. Assessment of Neurological Deficits and Seizure Activity

Seizure activity and neurological deficit were assessed at 4 and 24 h after tMCAO. Neurological deficit score was recorded for each rat as previously described [41, 42]. Neurological deficit findings were scored according to five-point scale: no neurological deficit = 0, forelimb flexion = 1, fore-limb flexion and reduced resistance to lateral push = 2; forelimb flexion, reduced resistance to lateral push and unilateral circling = 3; forelimb flexion, immotile or difficult to movement = 4. Seizure activity was classified using Racine's scale [43]: 0: no seizure was observed; 1: rhythmic mouth and facial movement; 2: rhythmic head nodding; 3: forelimb clonus; 4: rearing and bilateral forelimb clonus; 5: rearing and falling.

### 2.5. Measurement of Brain Infarct and Edema Volumes

Infarct volume evaluation was performed 24 h after tMCAO. Animals were deeply anesthetized and decapitated. Subsequently, their brains were removed and sectioned coronally into consecutive 2 mm thick slices. The series of slices from each brain was soaked in 2% 2,3,5-triphenyltetrazolium chloride (TTC) solution and incubated for 30 minutes at 37°C in a water bath. The slices were then transferred to 10% buffered formalin. Normal tissue appears red, whereas infarct tissue appears colorless.

Both sides of the TTC-stained slices were scanned using a flatbed scanner (Scanjet, Hewlett-Packard, USA) connected to a computer. Infarct areas in each slice were measured using Image J software (NIH). Then, infarct volume of each brain was determined by integration of the infracted areas of the slices [44]. Following formula was applied to eliminate the effect of swelling on the infarct volume: corrected infarct volume = infarct size × contralateral hemisphere size/ipsilateral hemisphere size. Cerebral infarct volume was measured as a percentage of the total brain, cortex, and striatum. Edema volume was also calculated by the following formula: edema = (volume of right hemisphere − volume of left hemisphere)/volume of left hemisphere.

### 2.6. Statistical Analysis

SPSS 11.5 software (SPSS, Chicago, Illinois) was used for the statistical calculations. One-way analysis of variance (for multigroup comparisons) was used for comparisons in infarct volume, brain edema, and testosterone serum levels followed by Bonferroni tests. Data on brain infarct volume and brain edema are reported as mean ± SD. Neurological deficit and seizure activity were determined using the Kruskal-Wallis test and are presented as medians and interquartile ranges (25th and 75th percentiles). Differences were considered to be statistically significant when *P* < 0.05.

## 3. Results

### 3.1. Testosterone Concentrations in Intact and Castrated Animals

**Figure 1:**
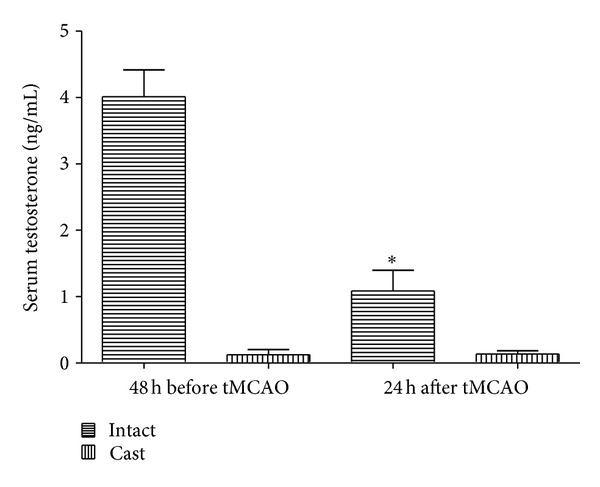
Serum testosterone concentrations (ng/mL) at 48 h before and 24 h after tMCAO in gonadally intact and castrated rats (mean ± SD). *Significant difference (*P* < 0.05) with intact group at 48 hours before tMCAO.

48 h before tMCAO serum testosterone concentrations were 4.01 ± 0.40 and 0.12 ± 0.07 ng/mL in intact and castrated groups, respectivel, (Figure 1). Serum testosterone levels 24 h after tMCAO were 1.08 ± 0.31 and 0.13 ± 0.05 ng/mL in intact and castrated groups, respectively. These data show testosterone concentration 24 h after tMCAO significantly reduced (*P* < 0.05) in gonadally intact group.

### 3.2. Infarct Volume and Brain Edema

**Figure 2:**
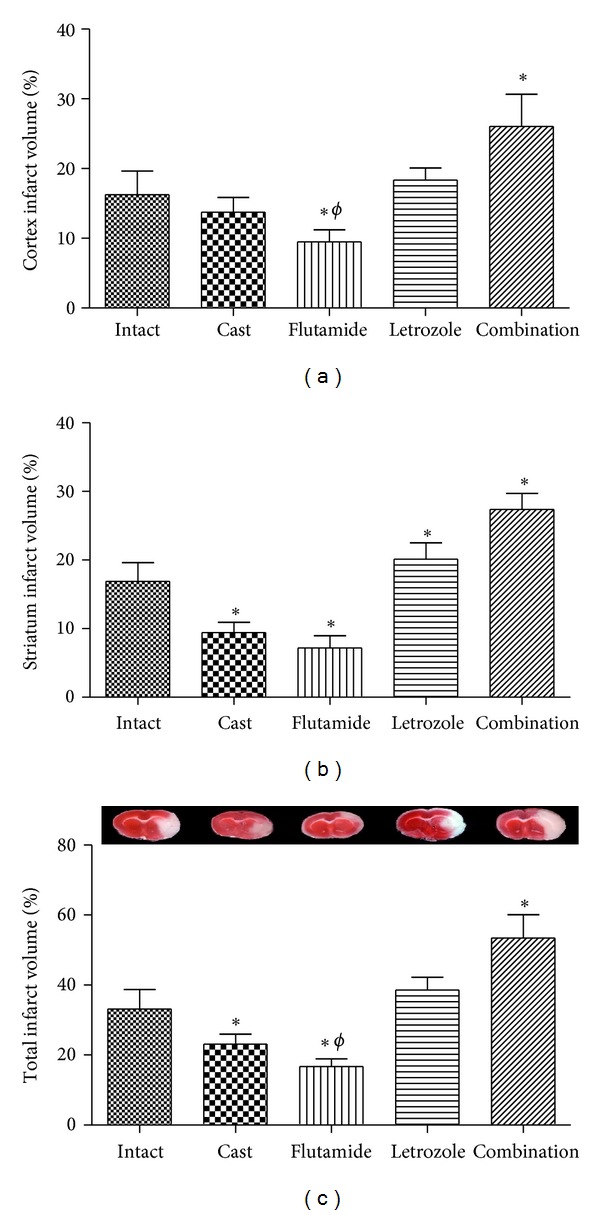
Cerebral infarct volume expressed as a percentage of the (a) cortex, (b) striatum, and (c) total brain in rats treated with castration, flutamide, letrozole, and flutamide in combination with letrozole (mean ± SD). *Significant difference (*P* < 0.05) with Intact. ^*ϕ*^Significant difference (*P* < 0.05) with Cast group.

24 h after 60 min tMCAO the brain injury was measured to the cortex and the striatum. The values for the cortical, striatal, and total infarct volume are shown in Figures 2(a), 2(b), and 2(c), respectively. Cortical infarct volume (Figure 2(a)) in the flutamide group was significantly reduced (9.4 ± 1.7; *P* < 0.05) compared to the intact (16.2 ± 3.4) and castrated (13.7 ± 2.1) groups, whereas cortical infarct volume in the combination group was significantly increased (26.0 ± 4.6; *P* < 0.05) compared to the intact group.

Striatal infarct volume (Figure 2(b)) in castrated and flutamide groups was significantly smaller (9.4 ± 1.4 and 7.1 ± 1.7, respectively; *P* < 0.05), whereas in letrozole and combinations groups was higher (20.1 ± 2.4 and 27.4 ± 2.3, respectively; *P* < 0.05) compared to the intact group (16.9 ± 2.8).

As shown in Figure 2(c), total infarct volume in castrated group was significantly smaller (23 ± 2.9; *P* < 0.05) than in intact group (33.1 ± 5.5). Moreover, flutamide group showed significant reduction (16.6 ± 2.2; *P* < 0.05) of total infarct volume compared to the intact and castrated groups. Combination therapy significantly increased (53.4 ± 6.7; *P* < 0.05) total infarct volume compared to the intact group.

**Figure 3:**
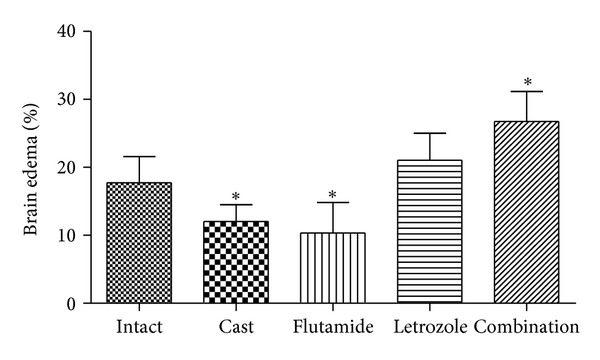
Brain edema expressed as a percentage of the total brain volume in rats treated with castration, flutamide, letrozole, and flutamide in combination with letrozole (mean ± SD). *Significant difference (*P* < 0.05) with Intact.

Brain edema results are shown in Figure 3. Castrated and flutamide groups showed significant reduction (12.0 ± 2.4 and 10.3 ± 4.4, respectively; *P* < 0.05) of brain swelling compared to the intact group (17.7 ± 3.8). Edema volume of combination therapy was significantly increased (26.7 ± 4.4; *P* < 0.05) compared to the intact group.

### 3.3. Neurological Deficits and Seizure Activity

**Figure 4:**
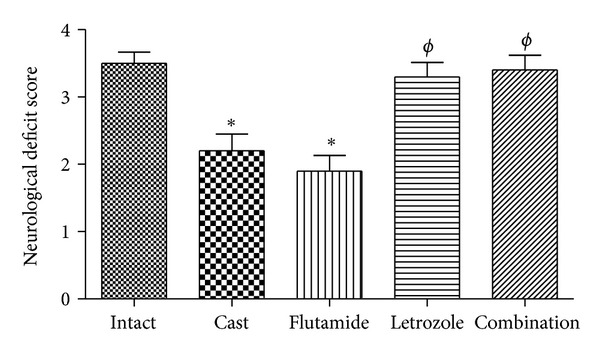
Neurological deficits scores at 24 h after tMCAO in rats treated with castration, flutamide, letrozole, and flutamide in combination with letrozole (mean ± SD). *Significant difference (*P* < 0.05) with Intact. ^*ϕ*^Significant difference (*P* < 0.05) with Cast group.

Neurological deficit score was evaluated at 4 and 24 h after tMCAO. There were no differences in neurological deficit scores between the experimental groups at 4 h after tMCAO (data not shown). Neurological deficit scores at 24 h after tMCAO (Figure 4) were significantly improved from 3.5 ± 0.52 in intact group to 2.2 ± 0.78 and 1.9 ± 0.73 in castrated and flutamide groups, respectively (*P* < 0.05). Furthermore, significant differences were found in neurological deficits at the letrozole (3.3 ± 0.67; *P* < 0.05) and the combination (3.4 ± 0.69; *P* < 0.05) groups than flutamide group.

**Figure 5:**
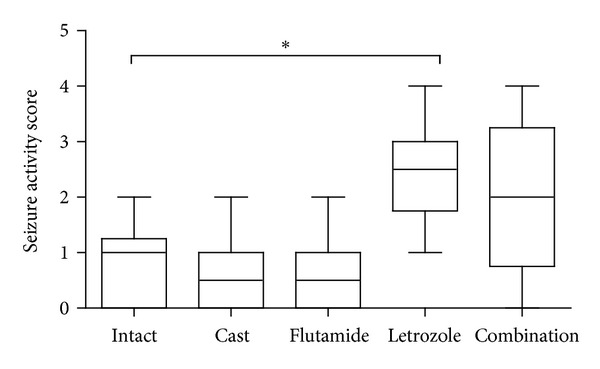
Seizure activity at 4 h after tMCAO in rats treated with castration, flutamide, letrozole and flutamide in combination with letrozole (mean ± SD). *Significant difference (*P* < 0.05) with letrozole group.

Seizure behaviors are shown in Figure 5. At 4 h after tMCAO the seizure activity was significantly increased in the letrozole group (2.4 ± 0.96; *P* < 0.05) only more than intact group (0.8 ± 0.78). No obvious seizure activity was observed in all experimental groups at 24 h after the tMCAO (data not shown).

## 4. Discussion

Clinical and epidemiological studies indicate that male sex is risk factors for stroke in humans [9, 10, 12]. This evidence was evaluated by earlier studies that mimic the pathology of human stroke and they proved that quantity of brain tissue injury after cerebral ischemia is greater in male versus female animals [9, 39]. Moreover, for testing effects of testosterone on cerebral ischemia, male rodents were castrated in order to testosterone depletion. In this model, castrated rodents after MCAO had lower infarct size than gonadally intact male animals, while testosterone administration to produce physiologic testosterone concentration in castrated rodents increases infarct size [17, 28, 30, 45]. In contrast, recent studies displayed that androgens have dose-dependent effects in cerebral ischemia, thereby testosterone in low physiological range gives protection while in high physiological range displays deleterious effects during an episode of MCAO [45, 46]. Whereas in real situation, plasma concentration of testosterone will decline slowly 24 h after cerebral ischemia and it has no constant concentration [26, 30, 47]. Therefore, to be close to the actual conditions of cerebral ischemia, we assessed the effect of testosterone via androgen receptor and aromatization to estrogen on cerebral ischemia by flutamide and letrozole pretreatment in gonadally intact rats that they subjected to tMCAO. So, it is suitable to focus on mechanisms of testosterone effects on stroke. Our results indicated that cortical infarct volume in castrated group was smaller than intact group, but this difference was not significant. While on the contrary, previous studies displayed castration can significantly reduces cortical infarct volume [28, 45]. Flutamide pretreatment significantly reduces cortical infarct volume compared to intact group and even castrated group. But other studies only indicated that flutamide reverses all testosterone effects (beneficial and deleterious) on infarct volume [45].

Castration significantly reduced striatal and total infarct volume as well as brain edema. Moreover, castration significantly improved neurological score. Although there was a similar tendency in cortical infarct volume of castrated rats, there was no significant difference with intact group. In agreement with our results, other studies have demonstrated that castration and subsequent testosterone depletion led to protection or no difference in infarct volume and better neurologic score after MCAO [17, 30, 45, 48]. On the other hand, they displayed testosterone administration in castrated rodents exacerbates infarct volume and neurologic score in part through amplifying of glutamate neurotoxicity in neuronal cells [19, 26, 49]. Therefore, it suggested that testosterone exposure could be harmful during the cerebral ischemia episode. Results of our study showed that flutamide significantly improved histological damage of rat brain in all regions and even flutamide had better effect than castration on infarct volume. In addition, flutamide confers protection on the brain edema and the neurologic score and although it had no significant difference with castrated group, but it was more effective than castration. On the contrary, most previous studies showed that testosterone confers deleterious effects to both adult castrates and gonadally intact rodents during stroke and these effects are abrogated by administration of the AR antagonist flutamide [45]. Also few recent studies indicated that maintaining plasma testosterone in low physiological range during MCAO induces protective effects in rodents and again this effect abolished by flutamide [19, 45].

Similar in vivo studies, both protective (in low physiological concentrations) and deleterious (in high physiological concentrations) effects of testosterone have been showed in glial and neuronal cultures under harmful conditions (e.g., oxidative stress, excitotoxicity, serum deprivation, and amyloid *β*) and again these effects were abolished by flutamide [19, 33–35, 37, 49, 50]. However, our study indicated flutamide had protective effects on gonadally intact rats during stroke. 

However, flutamide is commonly accounted pure antiandrogens without AR agonist activity [51, 52]. One explanation for the protective effects of flutamide in our work is that flutamide blocked deleterious effects or mimicked and enhanced neuroprotective effects of testosterone. In agreement with this explanation some studies in neuronal and glial cultures observed that flutamide failed to abolish neuroprotective effect of testosterone [35, 38, 53]. Furthermore, some works propose that flutamide did not only fail to reverse the effects of testosterone but mimicked or behaved as partial AR agonist for them as well [35, 38, 53, 54]. 

One interpretation of these data for our result is that flutamide is functioning as androgen agonist in activating cell signaling pathways that contributes to neuroprotection or as androgen antagonist in inhibiting cell signaling pathways that are active in deleterious effect of testosterone, or both of them. Another possible reason is that flutamide blocked AR and thereby increases available testosterone for conversion to estradiol by aromatase enzyme. Estradiol is known as a neuroprotective factor against cerebral ischemia and because aromatase is present in cerebral tissue and can convert testosterone into estradiol, an estrogen signaling pathway is reasonable [19, 30, 38, 45]. Although in our study letrozole increased significantly infarct volume only in striatum than intact group, it had similar tendency in cortex and total infarct volume as well as brain edema. On the other hand, letrozole significantly increased seizure activity. 

Brain aromatase is not only involved in the regulation of neuroendocrine events and behaviours connected with reproduction as previously thought, but also has roles in the reaction of brain tissue to damages and regulates synaptic plasticity, synaptic activity, and neurogenesis, via conversion of testosterone to estrogen [19, 55].

Other studies indicated that cerebral aromatase expression increased after brain injury in all damaged brain areas including, cortex, striatum, hippocampus, thalamus, and hypothalamus and exert neuroprotection effects via production of local estrogen [19, 55, 56]. On the other hand, wild-type rodents that were treated with aromatase inhibitors, as well as aromatase knockout (ArKO) rodents, showed more cortical and striatal damage in tMCAO model than wild-type animals treated with vehicle [19].

Also, an earlier study indicated that letrozole decreased estradiol levels, number of glutamic acid decarboxylase (GAD) positive cells, GAD expression as well as GABA production in hippocampal cultures, suggesting that aromatase by these effects in hippocampal neurons regulate synaptic activity [57]. According to our results, aromatase may play an important role in defense against overexcitation and seizure activity of neurons induced by glutamate after cerebral ischemia.

Coadministration of letrozole with flutamide showed that letrozole has a potency to reverse the beneficial effects of flutamide on histological damage and even make it worse than letrozole only level. One interpretation for effect of combination therapy is that letrozole ceases conversion testosterone to estrogen and thereby increases available testosterone to competing with flutamide for AR. On the other hand, the pernicious effect of letrozole could be related to seizures and not be related to testosterone levels. A previous study reported that early epileptic seizures worsen prognosis in human atherothrombotic stroke. This work stated that in-hospital mortality was significantly higher in stroke patients with early seizures [58]. These occurred in the temporal window, where a penumbra of potentially salvageable brain tissue is believed to exist and this may determine a greater area of definite cerebral infarction, which in turn would be associated with a poorer clinical course [58].

## 5. Conclusion

Contrary to the previous in vivo studies we have demonstrated that flutamide not only blocks deleterious effects of testosterone but also it can enhance neuroprotective effect of testosterone. Additional research is required to further describe the effects of flutamide on interaction between testosterone, aromatase, and AR during brain response to ischemia as well as determine which pathways are involved downstream of AR and aromatase. Clinically, our results suggest that flutamide and testosterone availability during cerebral ischemia may have beneficial effects in men. Finally, this work opens new encouraging perspectives for the protection of the brain from the ischemic injury. A prospective randomized trial of flutamide may be justified in humans.
